# Do Not Miss the (Genetic) Diagnosis of Gaucher Syndrome: A Narrative Review on Diagnostic Clues and Management in Severe Prenatal and Perinatal-Lethal Sporadic Cases

**DOI:** 10.3390/jcm10214890

**Published:** 2021-10-23

**Authors:** Aleksandra Jezela-Stanek, Grazina Kleinotiene, Karolina Chwialkowska, Anna Tylki-Szymańska

**Affiliations:** 1 Department of Genetics and Clinical Immunology, National Institute of Tuberculosis and Lung Disease, 01-138 Warsaw, Poland; 2 Faculty of Medicine, Vilnius University, 01513 Vilnius, Lithuania; grazina.kleinotiene@santa.lt; 3 Centre for Bioinformatics and Data Analysis, Medical University of Bialystok, 15-089 Bialystok, Poland; karolina.chwialkowska@umb.edu.pl; 4 Department of Pediatrics, Nutrition and Metabolic Diseases, Children’s Memorial Health Institute, 04-730 Warsaw, Poland; A.Tylki@ipczd.pl

**Keywords:** Gaucher disease, NIHF, perinatal-lethal Gaucher disease, PLGD, ichthyosis, GBA gene

## Abstract

With a growing number of proved therapies and clinical trials for many lysosomal storage disorders (LSDs), a lot of hope for many patients and families exists. However, there are sometimes cases with poor prognosis, fatal outcomes when our efforts must be directed towards a prompt and correct genetic diagnosis, which offers the only possibility of providing the family with appropriate prevention and treatment. To address this issue, in this article, we present the clinical and genetic hallmarks of the lethal form of Gaucher disease (PLGD) and discuss the potential management. We hope that this will draw attention to its specific manifestations (such as collodion-baby phenotype, ichthyosis, arthrogryposis), which differ from best-known GD complications and ensure appropriate diagnostic assessment to provide families at risk with reliable counselling and treatment to avoid the medical complication of GD.

## 1. Introduction

Gaucher disease (MIM # 230800) is one of the most common lysosomal storage disorders, characterized by an accumulation of glucocerebrosides resulting from mutations in the *GBA* gene (MIM *606463). The gene encodes a lysosomal membrane protein (glucocerebrosidase, GCase) that cleaves the beta-glucosidic linkage of glycosylceramide, an intermediate in glycolipid metabolism [1]. In the GD molecular etiology, a related pseudogene, located approximately 12 kb downstream of *GBA* on chromosome 1, also plays a role [2].

The disease is classically categorized phenotypically into three main types: non-neuronopathic type I, acute neuronopathic type II (GD2; # 230900), and subacute neuronopathic type III (GD3; # 231000). Among the clinical continuum of neuronopathic phenotypes, GD lethal form is also observed, which has a separate phenotype MIM number (**#** 608013) [2]. It is considered to be a distinct form of type II Gaucher disease. The prognosis for survival is decidedly poor in this GD form. Non-immune hydrops fetalis (NIHF), which is its key characteristic, is associated with death in utero with 90% risk or within two days of birth; in the absence of hydrops, death usually occurs within three months of life [3]. 

For the sporadic cases (in families with non-remarkable history), the earliest possible recognition of this disease is thus crucial as it allows for carrier screening, reliable genetic counselling and family planning. To facilitate the identification of the most severe types of GD, its perinatal lethal type (PLGD), particularly in the context of genetic testing, we aimed to present its molecular and clinical characteristics based on literature review and our own experience.

## 2. Materials and Methods

The cases included in our literature review have been identified through a literature (PubMed) search (by phrases: perinatal-lethal Gaucher disease; Gaucher disease AND prenatal) and encompass severe prenatal and perinatal-lethal genetically confirmed diagnoses of Gaucher disease. In the Discussion, we also referred to our cases.

The most recent review on the genetic etiology of non-immune hydrops fetalis (NIFH) has been published this year and included 23 cases of Gaucher disease [4]. Moreover, 10 other papers on the perinatal-lethal form of GD (not mentioned in the latest reviews: from 2008 [5] and from 2003 [6] have been identified.

In all these articles, molecular data have been reported in 2 and 10 papers, respectively, including 12 GBA variants, which were further analyzed for the purpose of our article.

*GBA* variants

*GBA* variants provided were classified according to ACMG/AMP guidelines (American College of Medical Genetics and Genomics and the Association for Molecular Pathology, Bethesda, Maryland, USA; Richards et al., 2015) with respect to current ACGS (The Association for Clinical Genomic Science, London, UK) and ClinGen (The Clinical Genome, National Institutes of Health—NIH, Bethesda, Maryland, USA) recommendations.

Variants were analyzed using hg38 human reference genome and MANE Selected transcript (NM_000157.4).

## 3. Results

Available clinical and molecular data on GD diagnoses performed in pregnancies complicated with non-immune hydrops fetalis (NIHF) and stillbirths and neonates presenting a potentially lethal form of GD are listed in Table 1.

## 4. Discussion

### 4.1. Genetic of Gaucher Disease

Up to date, more than 400 genetic mutations have been found to be associated with Gaucher disease (Gaucher Registry—International Collaborative Gaucher Group, Naarden, The Netherlands, 2021, accessed on 5 August 2021). Some of the variants are causing mild disease symptoms, while others are connected to very severe clinical phenotypes, characterized by the presence of primary neurologic disease.

The most common pathogenic genetic variant in *GBA* is NM_000157.4(NP_000148.2):p.(Asn409Ser), followed by c.84dupG, c.115+1G > A, and p.Leu483Pro. These four variants account for 50–60% of mutated alleles in non-Jewish individuals with type 1, non-neuronopathic GD. Homozygous individuals for p.Asn409Ser have a milder form of GD than people with just one copy plus another mutation or those having other pathogenic mutations. Individuals who are homozygous for the p.Leu483Pro variant tend to have disease connected to neurologic complications, which is a consequence of mutated protein dysfunctionality/residual glucocerebroside activity, and which may impact secondary protein structure as these residues differ in some properties. Functional studies of L483P indicate that it is poorly activated by phosphatidylserine, has a residual enzyme activity of 5–10% of wild type, and is unstable [19,20].

### 4.2. Genetic Characteristics of Provided Cases

Within the probands with prenatal or perinatal-lethal complications, presented in Table 1, none had any of the three most common mutations associated with GD: p.Asn409Ser, c.84dupG and c.115+1G > A. Variant p.Leu483Pro has been identified in one individual, however with compound heterozygosity with very rare pathogenic variant p.Pro440Leu (NC_000001.11:g.155235750G > A (dbSNP rsID: rs74598136) NM_000157.4:c.1319C > T NP_000148.2:p.(Pro440Leu)0. This variant can be found in the literature also as P440L, P391L, P353L and P401L.

Variant p.Pro440Leu was classified according to ACMG/AMP guidelines as pathogenic (criteria applied: PS4 + PM3 + PM1 + PM2_Supporting + PP3 + PP4). That is specifically based on the fact that it is:

- located in a mutational hot-spot in functional protein domain: Glycosyl hydrolase family 30 TIM-barrel domain (pfam; PM1),
- affected nucleotide position is conserved (GERP RS = 4.0399),
- predicted to affect protein function based on numerous in-silico predictors: metapredictor REVEL—Pathogenic (0.896); MutationTaster—Disease causing (1.0); SIFT—Damaging (0.001); PolyPhen-2 HumVar—Probably damaging (0.997); FATHMM-MKL—Damaging (0.9902); EIGEN—Pathogenic (0.7286) (PP3),
- absent from population controls (based on GnomAD v2.1.1 controls; PM2_Supporting)
- identified in multiple probands with GD clinical phenotype in trans with other pathogenic variant (PP4, PM3; PS4).

For comparison, the most frequent *GBA* pathogenic variant—p.(Leu483Pro (NC_000001.11:g.155235252A > G (dbSNP rsID: rs421016) NM_000157.4:c.1448T > C NP_000148.2:p.(Leu483Pro)) can be found in the literature as: L483P, L396P, L434P, L444P. It was classified according to ACMG/AMP guidelines as pathogenic, based on criteria applied: PS3 + PM1 + PM3 + PM5 + PP3 + PP4):

- change at amino acid residue where a different missense change (p.Leu483Arg) was determined to be pathogenic accordingly to ACMG guidelines (PM5),
- functional studies show a damaging effect on the protein function; PS3 [21,22]—has a residual enzyme activity of 13% of wild type, unstable, poorly activated by phosphatidylserine ([20]),
- located in a mutational hot-spot in functional protein domain: Glycosyl hydrolase family 30 beta-sandwich domain (pfam; PM1),
- affected nucleotide position is semi-conserved (GERP RS = 3.16),
- predicted to affect protein function based on numerous in-silico predictors: metapredictor REVEL—Pathogenic (0.8579); MutationTaster—Disease causing (1.0); SIFT—Damaging (0.002); PolyPhen-2 HumVar—Probably damaging (0.976); FATHMM-MKL—Damaging (0.9181) (PP3),
- present in reasonably low frequency in population controls (0.12% based on GnomAD v2.1.1 controls; PM2 not applicable),
- identified in multiple probands with GD clinical phenotype in the homozygous or compound heterozygous state with another pathogenic variant (PP4; PM3; PS4 not applicable due to frequency in the population).

Considering the severe, pre- and perinatal manifestation of Gaucher disease, the most interesting is another *GBA* variant—Rec*Nci*I allele, which is most frequently observed in the analyzed group. It is a name for a variant NC_000001.11:g.155235252A > G; 155235217C > G;155235203C > G (dbSNP rsIDs: rs421016, rs368060, rs1135675) NM_000157.4:c.1448T > C;1483G > C;1497G > C NP_000148.2:p.(Leu483Pro);(Ala495Pro);(Val499=) that can be classified according to ACMG/AMP guidelines as pathogenic because being a haplotype contains NM_000157.4:c.1448T > C NP_000148.2:p.(Leu483Pro) already classified as pathogenic (described above). Rec*Nci*I allele is a recombinant allele covering a complex triply mutant haplotype. This variant results from a gene conversion event between the functional *GBA* gene and its pseudogene *GBAP* located downstream [23]. Recombination is possible because *GBA* and its pseudogene are highly homologous—*GBAP* has 96% exonic sequence homology to the *GBA* coding region [24,25]. Close localization of such similar homologous regions increases the risk for recombination events giving rise to complex alleles. The homology between the *GBA* gene and its pseudogene is highest between exons 8 and 11, and thus most of the pathogenic mutations have been accumulated in this location [25]. Díaz-Font et al. [23] have proved that Rec*Nci*I alleles are generated by gene conversion, and they mapped the precise crossover site on the rearranged alleles [23]. Rec*Nci*I haplotype has been identified in multiple individuals with GD clinical phenotype in the homozygous or compound heterozygous state with another pathogenic variant [25], Table 1.

The patient described by Sudrié-Arnaud et al. [8] had homozygous deletion p.[(Gly39_Gln536del)] resulting in the complete loss of the region encoding exons from 3 to the 11—up to the protein end. The remaining gene part encoded properly only the first 38 amino acids; however, due to the absence of the rest of the native transcript, it can be supposed that mRNA is subjected to nonsense-mediated decay (NMD). Nonetheless, as the precise breakpoints are not known, the effect cannot be precisely predicted. In this patient, glucosylceramidase activity should have been completely absent.

Patient characterized by Akdag et al. [12] had homozygous c.1505G > A variant potentially resulting in simple missense p.Arg502His change; however in silico analyses revealed that this G > A change causes 5′ splicing donor loss and creation of a cryptic site 12 nucleotides downstream (VarSEAK, SpliceAI, dbscSNV ADA and RF scores). This is predicted to result in Arg502Gln and insertion of codons for 4 amino acids, preserving the reading frame. The resultant protein is 4 amino acids longer and has an insertion in the glycosyl hydrolase family 30 beta-sandwich domain, affecting its functioning.

### 4.3. Perinatal Lethal GD Complications and Management Options

Unfortunately, there is no definitive treatment available now for perinatal lethal complications when fetal ascites, hydrops, and/or hepatomegaly are prenatally diagnosed. Only symptomatic management can be considered, i.e., in fetal anemia (suspected based on middle cerebral artery peak systolic velocity, MCA-PSV and placentomegaly), transfusions are an option [15]; occasionally, in case of poly- or oligohydramnios (mentioned in Table 1) only symptomatic treatment is available. Otherwise, the pregnancy needs to be monitored, and optimal delivery time has to be planed. Depending on the legal regulations in the given country concerned, parents must be presented with options on how to proceed with the pregnancy so that they can make individual decisions. However, regardless of their decisions, during an obstetric assessment, the crucial step is to isolate fetal DNA (during amniocentesis, cordocentesis or from formalin-fixed paraffin-embedded (FFPE) blocks) to enable a genetic diagnosis to be made. The recommendation refers to every unexplained potentially lethal situation, especially in families with positive history (previous stillbirths, fetal edema, parents’ consanguinity). When, e.g., fetal hydrops is observed, we are unable to make any clinical diagnosis. Considering only inherited metabolic disease, it may be a feature of several, such as mucopolysaccharidosis (especially type VII, type IVA), galactosialidosis, infantile sialic acid storage disease, Gaucher disease 2 and 3, GM1 gangliosidosis, sialidosis or Niemann–Pick disease [4].

As presented in Table 1, the severe GD form usually manifests in the neonatal period with small birth weight, massive hepatosplenomegaly and ascites. In many cases, akinesia or joints contractures were described [8,13,15,16]. Decreased spontaneous movements at birth, followed by hypertonia and progressive neurologic deterioration, can be expected. The prognosis is now considered to be very poor, even in the attempt of early enzymatic therapy [18]. The neonates manifest significant respiratory distress, especially when lung hypoplasia and cardiomegaly are present, which altogether is unresponsive to any medical interventions. Maybe children with GD lethal form will benefit from chaperone therapy? To our knowledge, it has not yet been described. We can speculate that, as chaperones were proved to assist in the refolding of a mutated enzyme (i.e., GCase), significantly increased GCase activity in cultured macrophages derived from patient blood monocytic cell (PBMC) [26] and may pass through the blood-brain barrier thus restoring GCase activity in neurons [27].

Notably, the very characteristic and frequent feature is dermatological manifestations of PLGD, encompassing collodion-baby phenotype, sometimes accompanied by recognizable facial features (ectropion, everted lips), ichthyosis or ichthyosiform erythroderma (see Table 1). Its exact mechanism is still to be elucidated, but clinically these are unique and sporadic features that are certainly memorable. We can draw on our own experience here, when two cases were diagnosed with GD mainly because of severe skin manifestation (ichthyosis and subcutaneous hydrops). The presence of arthrogryposis and ichthyosis in newborns with hepatosplenomegaly should raise suspicion of GD. Therefore, we recommend that any biological material be stored to perform genetic tests or enzymes studies, necessary for genetic counselling regarding following pregnancies and family risk. Carriership of the disease in the parents can, in turn, only be confirmed by genetic testing; the results of enzyme analyses are not reliable.

### 4.4. Pre- and Perinatal Diagnostics of Lethal GD

Unfortunately, the available data concerning family histories, shown in Table 1, is limited, but we can note that even in consanguineous couples or history of pregnancy complications, the parents’ carriership has not been mentioned, and was established following the diagnosis of GD in an affected child. Yet, the options of prenatal diagnostics depend on the family history and genetic status of the parents. In a pregnancy at increased risk, when both pathogenic variants in a family are known, prenatal diagnosis is possible using molecular genetic testing. Otherwise, if only one or neither pathogenic variant in the family at risk is known, an assay of glucocerebrosidase enzymatic activity in the amniotic fluid can be performed. The use of prenatal testing is a personal decision of the parent(s), and discussion of these issues should always be offered. When the family history of GD is negative, the disease can only be diagnosed based on clinical suspicion and necessitates glucocerebrosidase enzymatic measurement and/or molecular testing of the entire *GBA* gene. The biochemical analyses allow the establishment of the diagnosis of GD but are, however, of no value for the parents as far as carriership is concerned.

Unfortunately, there is no definitive treatment available now for perinatal lethal complications when fetal ascites, hydrops, and/or hepatomegaly are prenatally diagnosed. Only symptomatic management can be considered, i.e., in fetal anemia (suspected based on middle cerebral artery peak systolic velocity, MCA-PSV and placentomegaly), transfusions are an option [15]; occasionally, in case of poly- or oligohydramnios (mentioned in Table 1) only symptomatic treatment is available. Otherwise, the pregnancy needs to be monitored, and optimal delivery time has to be planed. Depending on the legal regulations in the given country concerned, parents must be presented with options on how to proceed with the pregnancy so that they can make individual decisions. However, regardless of their decisions, during an obstetric assessment, the crucial step is to isolate fetal DNA (during amniocentesis, cordocentesis or from formalin-fixed paraffin-embedded (FFPE) blocks) to enable a genetic diagnosis to be made. The recommendation refers to every unexplained potentially lethal situation, especially in families with positive history (previous stillbirths, fetal edema, parents’ consanguinity). When, e.g., fetal hydrops is observed, we are unable to make any clinical diagnosis. Considering only inherited metabolic disease, it may be a feature of several, such as mucopolysaccharidosis (especially type VII, type IVA), galactosialidosis, infantile sialic acid storage disease, Gaucher disease 2 and 3, GM1 gangliosidosis, sialidosis or Niemann–Pick disease [4].

The genetic diagnosis of GD is complicated by the presence of a highly homologous pseudogene, *GBAP*; thus, the appropriate genetic test must be considered; PCR-based methods have to be designed to differentiate *GBA* from the pseudogene. Some diagnostic gene panels may possibly not include the *GBA* gene. Moreover, testing for the p.Leu483Pro variant alone will not distinguish its isolated presence from Rec alleles [28].

Another concern is the fact that the number of rare genetic variants known to date in the *GBA* gene is very high, and none of the most frequent variants has been identified in fetal cases presented in Table 1 fatal GD cases. Thus, because the probability that these variants are present in the proband with pre- and perinatal characteristics of Gaucher disease is extremely low, the laboratory analyses should not be limited only to the most popular mutations present in a given population. At that point, without doubt, the Rec*Nci*I recombinant allele should be taken into account, as noted in half of our reviewed cases (6/12).

## Figures and Tables

**Table 1:** *GBA* variants identified in Gaucher disease based on prenatal or perinatal-lethal complication.

Pathogenic Variant (as Presented in the Article)	HGVS cDNA—NM_000157.4 Protein—NP_000148.2	Prenatal/Perinatal Features and Weeks’ Gestation (WG)	Outcome	Family History, Ethnicity	Reference
		prenatal NIHF as the main feature			
[p.P391L] + [p.L444P]	c.[1319C > T];[1448T > C] p.[(Pro440Leu)];[(Leu483Pro)]	at 26 WG: hydrops fetalis, no fetal motility, moderate cardiac dysfunction and reduced size of thorax	hydropic fetus, splenomegaly, pulmonary hypoplasia, micropenis with no malformations	nd	[7]
homozygous deletion: NM_001005741.2: c.(115+1_116−1) _(1616+1_?)del	c.[(115+1_116−1) _(1611+1_?)del];[c.(115+1_116−1) _(1611+1_?)del] p.[(Gly39_Gln536del)];[(Gly39_Gln536del)]	at 23 WG: microcephaly, ascites, and fetal akinesia; at 24 WG: hydrops fetalis and pontocerebellar hypoplasia in addition to microcephaly	symmetric growth restriction with all biometric parameters < 5th percentile; diffuse cutaneous “collodion-like” edema, nonspecific craniofacialdysmorphism related to microcephaly, macroglossia, arthrogryposis, as well as major hepatosplenomegaly and ascites the brain shows no primary fissures associated with the brainstem and vermis hypoplasia.	the previous child in the family was born to the first-degree consanguineous parents	[8]
	perinatal lethal manifestation				
c.[1505+1_1505+12ins; 1505 A > G]/RecNciI	c.[1505G > A](predicted to disrupt splicing and resulting in: c.1505+1_1505+12ins;1505G > A);[1448T > C;1483G > C;1497G > C] p.[(Arg502His)](predicted to disrupt splicing and resulting in p.Arg502Gln;Gln502ins4);[(Leu483Pro);(Ala495Pro);(Val499=)]	polyhydramnios	from birth:non-immune hydrops fetalis and hepatosplenomegaly, collodion skin, dysmorphic features (low-set malformed ears, hypertelorism, narrow palpebral fissures, a flat occipital bone, bell-shaped thorax with extremely thin ribs, short neck, and small scrotum); died at 14 days of life	Greek case	[9]
RecNciI allele (L444P, A456P and V460V); p. R131C (c.508 C > T)	c.[1448T > C;1483G > C;1497G > C];[508C > T] p.[(Leu483Pro);(Ala495Pro);(Val499=)];[(Arg170Cys)]	30 WG: hepatosplenomegaly	at birth:generalized skin edema and extensive peeling of skin cardiomegaly tonic seizures profound and persistent metabolic acidosis; died at 6 h of life	nonconsanguinous Asian mother	[10]
compound heterozygosity for R131C and RecNciI (A456P (cDNA 1483G.C, genomic DNA 7354), and V460V (cDNA 1497G.C, genomic DNA 7368)	c.[508C > T];[1448T > C;1483G > C;1497G > C] p.[Arg170Cys];[(Leu483Pro);(Ala495Pro);(Val499=)]	18 WG: isolated choroid plexus cysts 30 WG: unremarkable 35 WG: hepatosplenomegaly, with both organs measuring above the 95th percentile 37 WG: also decreased fetal movements	at birth: bradycardia, apnea, and hypertonia generalized edema, ichthyotic and collodion skin, palpable hepatosplenomegalypoor biventricular function with pulmonary hypertension, transverse arch flow reversal, and a large patent ductus arteriosus with a right-to-left shunt; died 6 h after birth	nonconsanguineouscouple of Chinese ancestry	[11]
homozygous R463H	c.[1505G > A];[1505G > A](predicted to disrupt splicing and resulting in: c.[1505+1_1505+12ins;1505G > A];[1505+1_1505+12ins;1505G > A]) p.[(Arg502His)];[(Arg502His)](predicted to disrupt splicing and resulting in p.[Arg502Gln;Gln502ins4)];[(Arg502Gln_Gln502ins4]		34 WG: cesarian sections, severe neurologic signs with refractory thrombocytopenia, hepatosplenomegaly; tight and shiny skin; desquamating on the wrist and ankle, multiple dysmorphic features, including microphthalmia, a flattened nasal bridge, anteverted nares, a short throat, and a partial Simian crease; seizures did not respond to multiple anticonvulsantstherapy;died at 46 days of age from respiratory failure	consanguinity was reported among the parents but there was no pertinent family history related to childhood disease or death	[12]
homozygosity for the RecNciI allele (c.1448T > C, c.1483G > C and c.1497G > C)	c.[1448T > C;1483G > C;1497G > C];c.[1448T > C;1483G > C;1497G > C] p.[(Leu483Pro);(Ala495Pro);(Val499=)];p.[(Leu483Pro);(Ala495Pro);(Val499=)]	30 WG: foetal akinesiasubsequently: progressive hepatosplenomegaly, cerebellar hypoplasia, pulmonary hypoplasia and unusual facial features	at birth: ichthyosis and diffuse purpural rash over most of the body facial dysmorphism (flattened-face, hypertelorism, retrognathia, anteverted nares, everted lips and ankyloblepharon) flexion contractures, thin gracile ribs with occasional gaps and abnormal phalanges in the hands; lungs hypoplasia with features of hepatosplenomegalyhypoplastic cerebellum with atrophic pons atypical macrophages within the brain; died 2 h after birth	parents are second cousins	[13]
c.1255G > A leading to the substitution of Aspartic Acid by Asparagine (p.Asp419Asn) [no data on homozygosity]	c.1255G > A p.(Asp419Asn) *zygosity unknown*	third trimester: severe hydrops fetalis with skin edema, polyhydramnios, hepatomegaly, clustered bowel loops, and fetal hypokinesia.	at birth: apnea shiny and thickened skin, reminiscent of a collodion-baby phenotype died in the first day of life	previous preterm male stillborn and undiagnosed; non-immune hydrops fetalis cases of non-immune hydrops fetalis	[14]
homozygosity for the RecNcil mutation	c.[1448T > C;1483G > C;1497G > C];c.[1448T > C;1483G > C;1497G > C] p.[(Leu483Pro);(Ala495Pro);(Val499=)];p.[(Leu483Pro);(Ala495Pro);(Val499=)]	27 WG: severe fetal hydrops with increased abdominal circumference due to ascites and elevated Middle Cerebral Artery Peak Systolic Velocity fetal anemia (treated with transfusions) 28 WG: intrauterine death	subtle facial anomalies including a high arched palate with no clefting, flat, broad nose with hypertelorism, and rounded face stiff elbow and knee joints with fixed flexion deformities and pterygia on the flexor surfaces	east Indian ethnic background;previous uninvestigated male stillbirth followed by an uncomplicated pregnancy	[15]
missense G234E and H413P heterozygous mutations	c.[701G > A];[1238A > C] p.[(Gly234Glu)];[(.His413Pro)]	36 WG: oligohydramnios increased cardiothoracic ratio, and a small lung volume, indicating pulmonary hypoplasia	at birth: severe respiratory distress flexion contractures at the elbow and knee joint, hypertonia, akinesiahepatosplenomegalyfacial dysmorphism (hypertelorism, downslanting eyes, an eye movement disorder, ectropion, hypophysis, thickening of the helix, constriction of the auricular rim, curl of the auricle and auricle cartilage, a flat nasal bridge, small nostrils, and everted lips) ichthyotic and collodion skin covered the entire body hypoplastic external genitals myoclonic seizure	Chinese mother (gravida 2, para 2); non-consanguineous parents	[16]
c.667T > C p.W223R; c. 1448C > T p. L483P (RecNcI)	c.[667T > C];[1488C > T] p.[(Trp223Arg)];[(Leu483Arg)]	28 WG: NIHF, hepatosplenomegaly	29–30 WG: intrauterine fetal demise— NIHF, facial dysmorphism, hepatosplenomegaly, cerebellum and pons hypoplasia	GI—miscarriage GII—fetal edema (NIHF), splenomegaly at 29 WG; boy died 15 min after birth	[17]
p. Asp448His (NM_ 000157.3:c.1342G > C) and p.Tyr531Ter (NM_000157.3:c.1593C > A).	c.[1342G > C];[1593C > A] p.[(Asp448His)];[(Tyr531Ter)]	polyhydramnios	at birth: widespread blueberry muffin skin lesion s and respiratory distress hepatosplenomegaly and cardiomegalyanemia and thrombocytopenia prompt initiation of enzyme replacement therapy clinical condition progressively worsened, leading to death at 3 months of age due to hepato-renal insufficiency	nd	[18]
